# The pattern-based interpretation of p53 immunohistochemical expression as a surrogate marker for *TP53* mutations in colorectal cancer

**DOI:** 10.1007/s00428-024-03790-z

**Published:** 2024-03-21

**Authors:** Mitsumasa Osakabe, Noriyuki Yamada, Ryo Sugimoto, Noriyuki Uesugi, Eiichi Nakao, Michitaka Honda, Naoki Yanagawa, Tamotsu Sugai

**Affiliations:** 1 Department of Molecular Diagnostic Pathology, School of Medicine, Iwate Medical University, 2-1-1, Shiwagun’yahabachou, Morioka, 028-3695 Japan; 2 Diagnostic Pathology Center, Southern Tohoku General Hospital, 7-115, Hachiyamada, Kooriyama City, 963-8563 Japan; 3 Department of Minimally Invasive Surgical and Medical Oncology, Fukushima Medical University, 1 Hikarigaoka Fukushima, Fukushima, 960-1295 Japan; 4 Department of Surgery, Southern Tohoku General Hospital, 7-115, Hachiyamada, Kooriyama City, 963-8563 Japan

**Keywords:** p53 immunohistochemistry, *TP53* mutation, Overexpression, Null type, Cytoplasmic pattern, Colorectal cancer

## Abstract

Mutations in the *TP53* gene, most commonly observed in colorectal cancer (CRC), play an essential role in colorectal carcinogenesis. Although p53 immunohistochemical (IHC) expression patterns have been argued to serve as an excellent surrogate marker for *TP53* mutations, its performance has not been confirmed in CRC. We aimed to determine whether p53 IHC expression patterns accurately predict *TP53* mutation status as examined by next-generation sequencing (NGS). We performed p53 IHC and sequencing of *TP53* by NGS in 92 CRC cases with a microsatellite stable phenotype to investigate the correlation between *TP53* mutation status and p53 IHC expression. The concordance between p53 IHC and *TP53* mutation was 84/92 (91.3%) overall. However, 6 mutant cases were found in 39 cases with a wild-type IHC pattern. Additionally, there were two discordant cases in which an abnormal p53 IHC pattern (overexpression or cytoplasmic pattern) was found, while NGS detected wild-type p53. Therefore, the optimized p53 IHC performs well and serves as a surrogate test for *TP53* mutation in CRC cases. Furthermore, it demonstrates excellent reproducibility between two independent experienced pathologists and may have novel clinical utility for molecular classification algorithms in CRC. We suggest that the four-tier classification of p53 IHC patterns is helpful to evaluate molecular colorectal carcinogenesis.

**Supplementary Information:**

The online version contains supplementary material available at 10.1007/s00428-024-03790-z.

## Introduction

Colorectal cancer (CRC) is one of the most common cancer types worldwide [1]. *TP53* mutations are present in > 50 ~ 75% of CRC cases and are an early driver molecular event [2]. Therefore, *TP53* mutations may play a major role in carcinogenesis [3]. *TP53* mutations are the most important molecular factor involved in various cancer types, including gastric, esophageal, colorectal, ovarian, and cervical carcinomas [4, 5]. In addition, although many studies have shown that *TP53* mutations are associated with worse prognosis [7, 8], its significance for clinical practice remains unknown. Therefore, *TP53* mutation status is currently not considered for clinical decision making.

P53 immunohistochemistry (IHC) is widely used as a surrogate marker for *TP53* mutation testing in diagnostic gastrointestinal pathology, particularly for evaluating CRC [9, 10]. The most common diagnostic application in colorectal neoplasia is to distinguish colorectal adenoma with low-grade dysplasia from that with high-grade dysplasia [11]. However, there may be overlap in the IHC staining pattern between low-grade and high-grade dysplasia. Therefore, there is a limit to the application of p53 IHC staining in histological differentiation between these types of adenomas. In addition, pathologists have difficulty in interpreting p53 IHC patterns in routine practice, given that several IHC patterns have previously been reported to represent *TP53* mutational status, including overexpression, cytoplasmic, null-type, and wild-type patterns [12–14]. Recent studies have shown that a new four-tier classification of p53 IHC helps resolve the underlying pathogenesis of *TP53* mutations occurring in various cancer types [12–15]. It is challenging to distinguish each of the four IHC patterns, in particular the null-type from wild-type and the overexpression type from the cytoplasmic type. These p53 IHC patterns may have significant implications for clinical decisions, including pathological diagnosis, surgical treatment planning, adjuvant therapy selection, and risk assessment for hereditary syndromes [12–15]. Hence, p53 IHC has been widely used as a surrogate for *TP53* mutations in the pathological diagnosis of CRC and for response to therapy [12–15].

In this study, we determined the sensitivity and specificity of IHC to predict the *TP53* mutation status in CRC. We compared clinically relevant assays for p53 staining with next-generation sequencing (NGS) data of isolated tumor glands as the gold standard reference. The secondary aim of the study was to investigate misclassified cases to categorize *TP53* mutations with unexpected patterns of p53 staining in CRC.

## Materials and methods

### Patients

Tumor specimens were obtained from 92 patients with CRC with microsatellite stability (MSS) who had undergone colectomy at Iwate Medical University Hospital (Iwate, Japan) between 2015 and 2022. MSS was determined based on a previous report [16]. Clinicopathological variables, including age, sex, tumor location, tumor size, histological classification, and tumor stage, were recorded according to the Classification of the Japanese Society for Cancer of the Colon and Rectum [17] (Table 1). None of the patients received preoperative neoadjuvant therapy or radiotherapy.

**Table 1:** Clinicopathological characteristics of rectal cancer cases analyzed

Characteristics	Number of cases (%)	
Total	92	
Sex		
Male	34	(37)
Female	58	(63)
Age, years, median (range)	75 (42–93)	
Location		
C/A/T/D/S/R	14/30/10/4/17/17	
Size, mm, median (range)	50 (17–120)	
Histological type		
Well differentiated	24	(26.1)
Moderately differentiated	42	(45.7)
Poorly differentiated	26	(28.3)
Stage		
I	16	(17.4)
II	35	(38)
III	38	(41.3)
IV	3	(3.3)

C, cecum; A, ascending colon; T, transverse colon; D, descending colon; S, sigmoid colon; R, rectum

All patients provided written informed consent for participation in this study, and the study was approved by the Ethical Committee of Iwate Medical University (HG2021-023).

### Determination of sample size

We performed a pilot study in which we evaluated 30 CRC cases to determine the correlation between p53 IHC patterns and *TP53* mutation status. The statistical power of the study was set to 0.8, which is commonly used based on the “Cantor method” using a statistical package in R (the irr package) [18]. Finally, the sample size required to identify a correlation between p53 IHC patterns and *TP53* mutation status was determined using sample size package in R (version 4.3.1) to be minimally 86 cases.

### Crypt isolation method

We used a crypt isolation method to obtain only tumor DNA in accordance with previously reported methods [19]. In addition, tumor tissues for mutation analysis were obtained from a region of the resected colon adjacent to the site used for histological analysis. Crypt isolation was used for molecular analysis to avoid mixture with interstitial cells, which do not have molecular alterations present in tumor tissue. In addition, DNA quality extracted from isolated tumor glands is superior to that from paraffin-embedded tissue, as shown in our previous study [19]. Normal mucosa was taken from normal mucosa most distant from the cancer region. Briefly, fresh normal mucosa and tumor samples were minced and incubated at 37 °C for 60 min in calcium- and magnesium-free Hanks’ balanced salt solution (CMF) containing 30 mM ethylenediaminetetraacetic acid (EDTA). The tissue was then stirred in CMF for 30–40 min. The isolated crypts were immediately fixed in 70% ethanol and stored at 4 °C until DNA extraction. The fixed isolated crypts were observed under a dissecting microscope (SZ60; Olympus, Tokyo). Some isolated crypts were routinely processed for histopathological analysis to morphologically confirm their isolated nature. No contamination, such as interstitial cells, was observed in any of the 92 samples.

### DNA extraction

DNA from normal and tumor glands was extracted by standard SDS proteinase K treatment. DNA extracted from the samples was resuspended in TE buffer (10 mM Tris–HCl, 1 mM EDTA [pH 8.0]).

### Tissue microarray (TMA) construction

The tumor tissue used for p53 IHC staining was obtained from a region of the resected colon adjacent to the site used for crypt isolation, which was used for molecular analysis. The TMAs were assembled using a manual tissue array (Azumaya Co, Tokyo, Japan). Tissue cores of 5 mm were taken from each targeted lesion and placed into a recipient block containing 12 cores, including 10 cores for cancer tissues and 2 cores for control tissues (normal colon; CRC). After construction, 3 μm sections were cut and stained with hematoxylin and eosin on the initial slides to verify the histologic diagnosis. Serial sections were cut from the TMA block for immunohistochemical staining.

### P53 immunohistochemistry

**Fig. 1:**
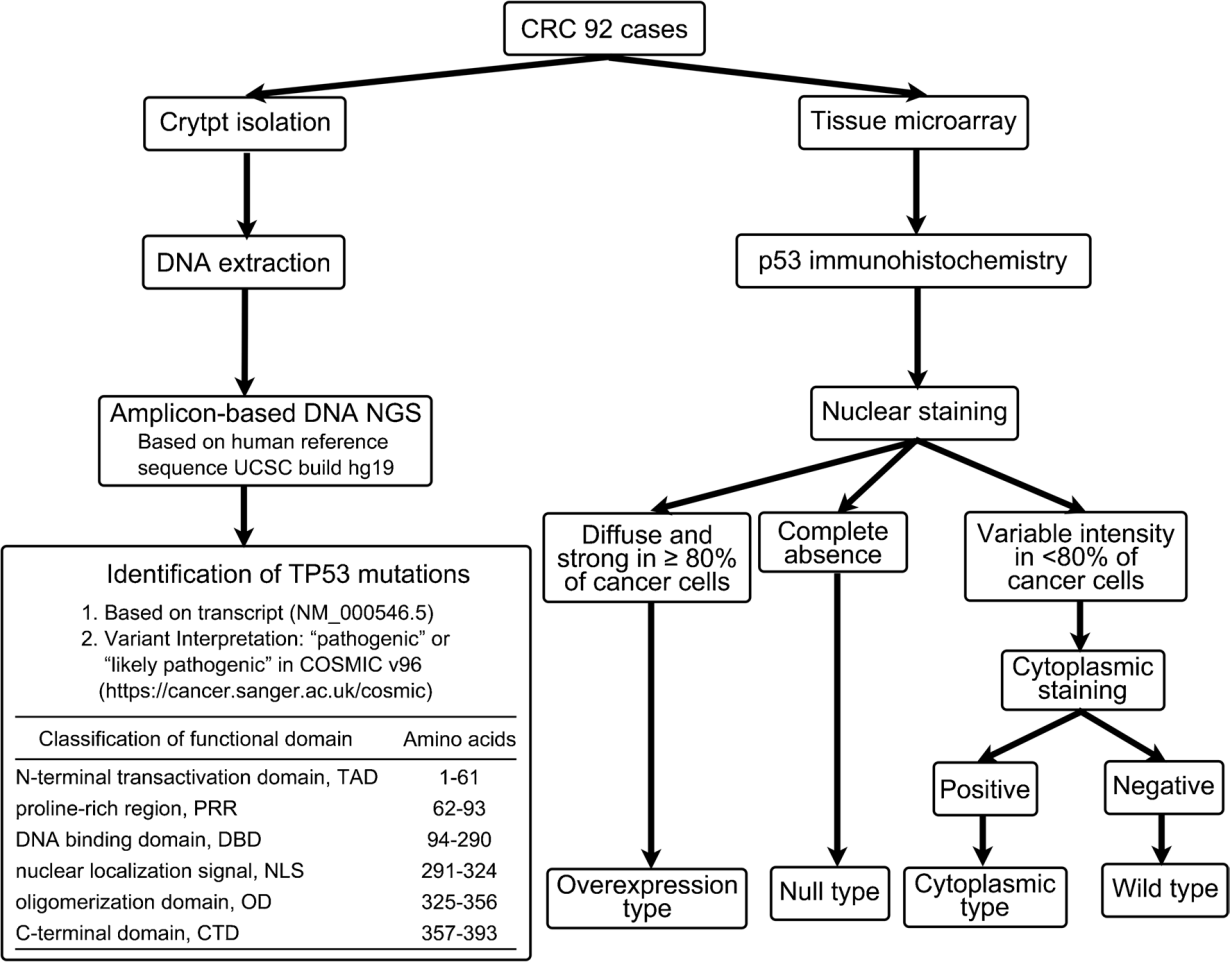
Algorithm for determination of p53 IHC patterns

P53 IHC was performed according to the NordicQC method (https://www.nordiqc.org) using a DAKO autostainer platform (DAKO autostainer Link48). P53 was visualized using the p53 antibody clone DO-7 (DAKO). P53 IHC staining patterns were evaluated by two independent, experienced pathologists using whole slide sections. The algorithm for interpreting p53 IHC is shown in Fig. 1. As a control for abnormal p53 staining, a CRC case with a p53 overexpression pattern with a known *TP53* mutation in the tumor tissue was used. We used normal appendiceal crypt tissue as the control for wild-type p53 staining as per NordicQC recommendation.

### Criteria for the evaluation of nuclear staining

If nuclear p53 staining was observed with uniformly strong, diffuse nuclear expression in at least 80% of tumor cells (regardless of whether cytoplasmic staining was present), the staining pattern was classified as “p53 overexpression pattern” [15]. No p53 staining (complete absence of any nuclear expression) was termed as “null-pattern” [15]. Next, if cytoplasmic staining was present in cases in which the nuclear staining did not meet the criteria for the p53 overexpression pattern or p53 null-pattern, the case was classified as “p53 cytoplasmic pattern” [15]. Finally, a staining pattern that did not meet any of the above criteria (weak staining pattern of nuclear or cytoplasmic staining), was classified as “p53 wild-type pattern” [15]. The initial assessment of IHC patterns was performed without knowledge of the *TP53* mutation status or the p53 IHC interpretation in the original diagnostic report. However, if we were aware of the initial interpretation of p53 IHC, we excluded the subclonal case. IHC findings were reassessed by two senior pathologists (MO and TS). Divergent opinions were discussed until a consensus was reached.

### Next‐generation sequencing (NGS)

Targeted NGS was performed on isolated tumor glands. In brief, NGS libraries were prepared using a custom panel (Illumina, San Diego, CA, USA) containing 753 amplicons covering 82 exonic regions across 28 genes. Sequencing was performed for each pool by loading 600 μL of library mixes. Sequencing analysis viewer software (SAV; Illumina) was used to confirm quality metrics with interop files along with run info and parameters. A Phred score of Q30 was considered for each run. MiSeq Reporter software (Illumina) was used for demultiplexing, sequence alignment, and variant calling. Successful sequencing runs generated a FASTQ file for each sample pool and a single genomic variant call file (VCF). Annotation of detected variants was performed using Illumina Variant Studio version 2.2 software (Illumina). Every variant with a variant allele frequency < 10% was filtered and excluded before review. Detected variants were marked with a PASS filter flag if the following criteria were met: the variant was present in each pool, the cumulative depth was 1000 × per pool, and the average depth was 500 × per pool. Variant classification was performed using ClinVar (http://www.ncbi.nlm.nih.gov/clinvar) and COSMIC (http://cancer.sanger.ac.uk/cosmic) databases. Pathogenic and likely pathogenic variants were reported according to standard guidelines.

### Sanger sequencing

The *TP53* coding sequences (exons 4–8) were amplified using a PCR-based sequencing, as described previously [18]. The PCR-based sequencing was performed for 10 cases in which there was a discordance between the final p53 IHC classification and NGS-determined *TP53* mutational status. *TP53* mutation sites were determined in *TP53* exons 4 to 8.

### Statistical analysis

We used JMP Pro 16.1 (SAS Institute, Cary, NC, USA) for statistical analysis. The diagnostic test performance of p53 IHC patterns was quantified by Cohen’s kappa for agreement, and the sensitivity and specificity accuracy of p53 IHC compared to *TP53* mutation status was calculated.

## Results

**Fig. 2:**
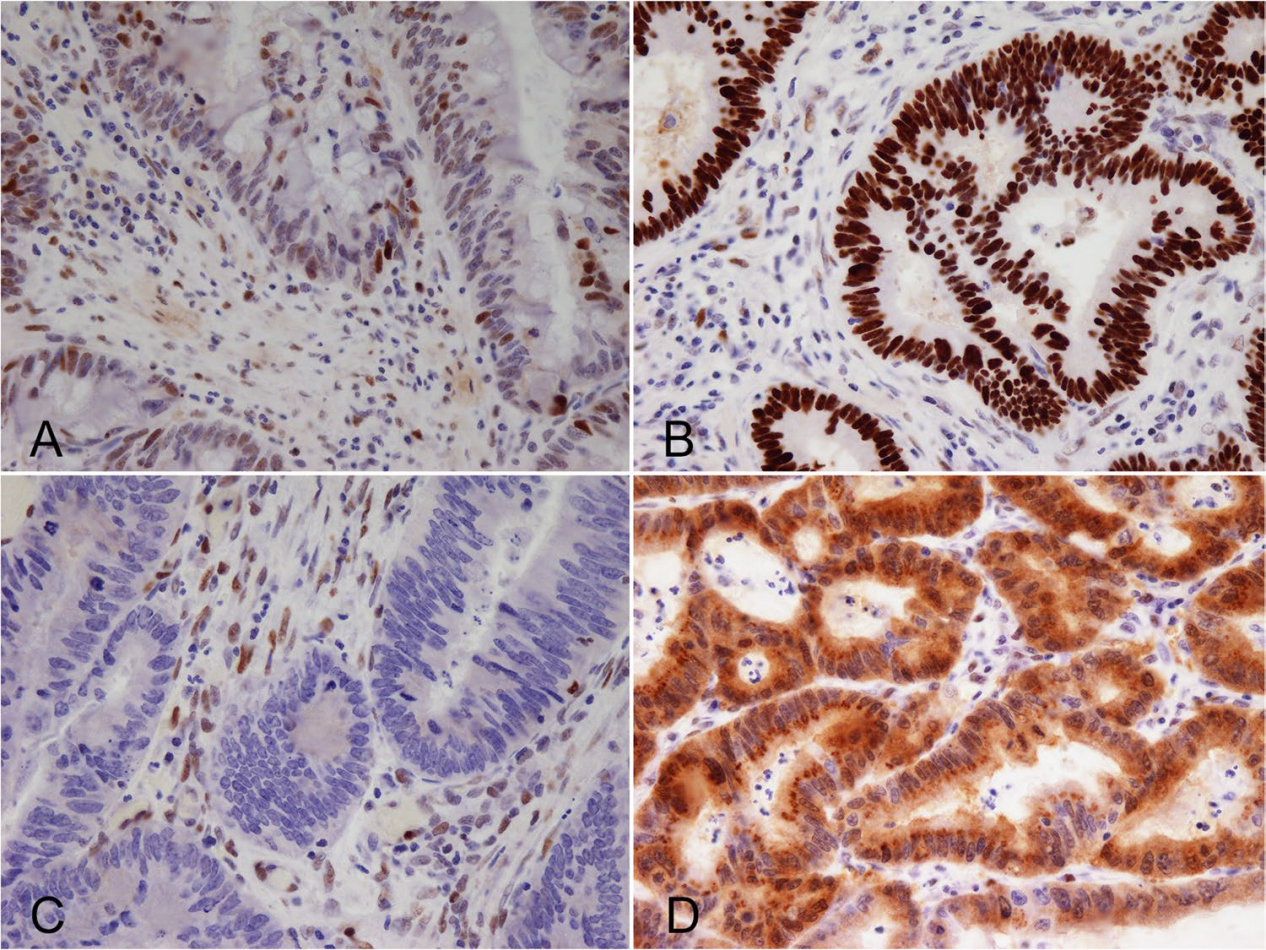
Different patterns of p53 expression. **A**. wild-type; **B**. overexpression; **C**. null-type; **D**. cytoplasmic pattern

We selected 92 CRC cases for *TP53* sequencing and p53 IHC, which were present on a TMA that had been subjected to detailed pathology review and immunophenotyping. The algorithm for the p53 IHC staining patterns scoring system is shown in Fig. 2.

### TP53 mutation analysis

The 92 isolated tumor glands were subjected to tagged-amplicon sequencing. The median sequencing depth for *TP53* was estimated as at least 1000 [IQR (interquartile range) 1150–3279] and the median *TP53* mutant allelic fraction was 0.37 (IQR 0.25–0.63).

**Fig. 3:**
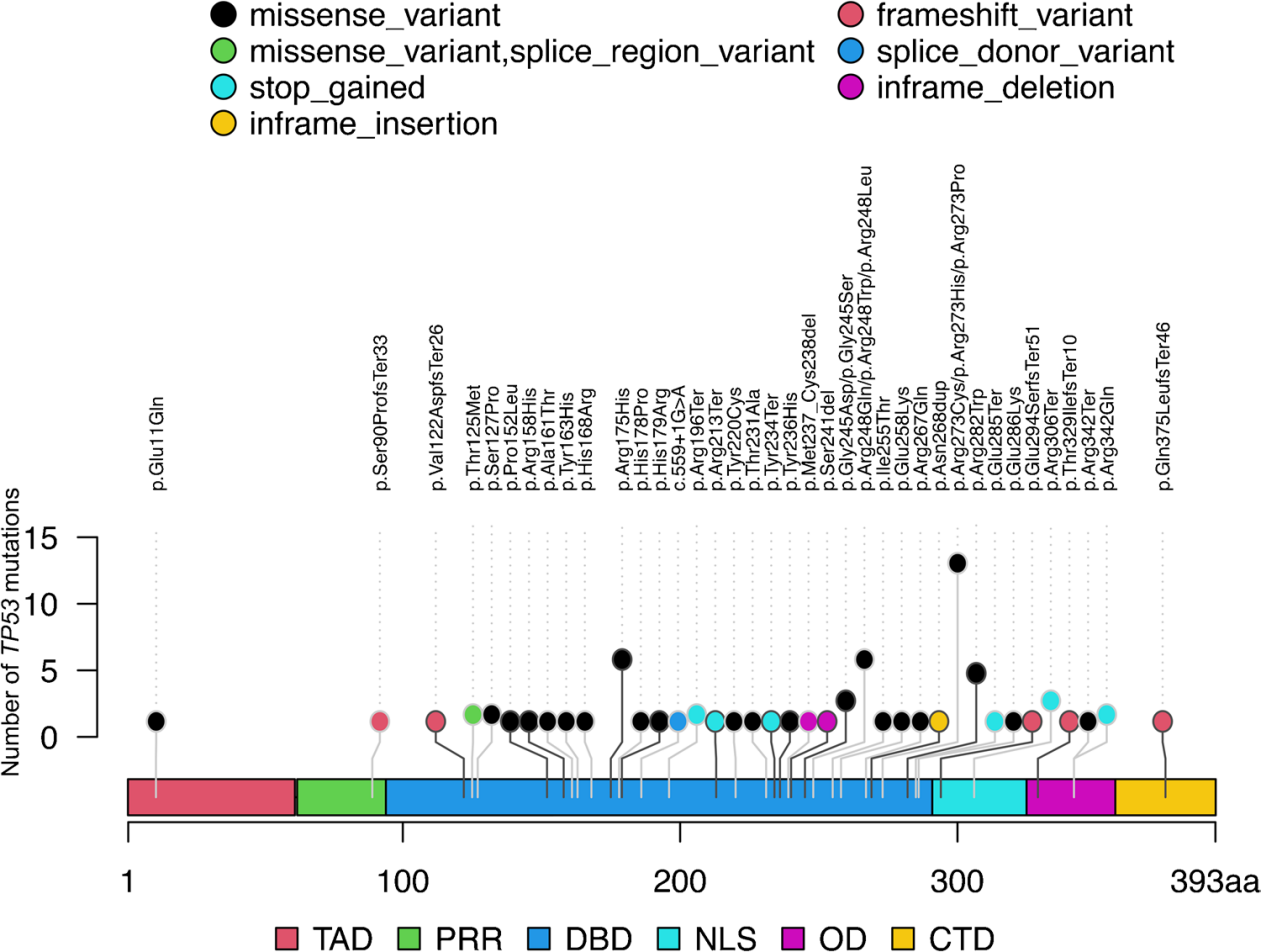
Frequency and position of *TP53* mutations. (a) Schematic of the *TP53* gene showing protein domains (open boxes) with lollipops showing positions and counts of identified mutations. Mutation type is indicated by circle fill: Black, missense; Green, missense-variant (splice variant); sky blue, stop-gained; yellow, inframe-insertion; orange, frame-shift variant; Blue, splice-donor variant; red, inframe-deletion; red square, TAD; green square, PRR; Blue square, DBD; sky blue square, NLS; red square, OD; yellow square, CTD. TAD, N-terminal transactivation domain; PRR, proline-rich region; DBD, DNA binding domain; NLS, nuclear localization signal; OD, oligomerization domain; CTD, C-terminal domain

*TP53* pathogenic mutations (non-synonymous mutation) in CRC were detected in 72 out of 92 cases (78.3%). The most frequently found mutations were missense variants (53/72, 73.6%), whereas a stop codon variant was detected in 10 of 72 variants (13.9%). Frameshift variants (n = 5), in-frame deletions (n = 2), in-frame insertion (n = 1), and splice donor variants (n = 1) were less common in the present study. Common amino acid substitutions were p.Arg273Cys (n = 8, 11.1%), p.Arg175His (n = 6, 8.3%), p.Arg282Trp (n = 5, 6.9%), p.Arg273His (n = 4, 5.6%) and p.Arg248Gln (n = 3, 4.2%). All mutations were deleterious *TP53* mutations due to the exclusion of non-pathogenetic mutations in the present study. We examined which *TP53* gene domains were associated with *TP53* mutations. The most frequent domain with *TP53* mutations was the DNA binding domain (61/72 variants, 84.7%), whereas fewer *TP53* mutation were found in the remaining domains [N-terminal transactivation domain, TAD (amino acids, AA1-61), N = 1 (1.4%); proline-rich region, PRR (AA62-93), N = 1 (1.4%); nuclear localization signal, NLS (AA291-324), N = 4 (5.6%); Oligomerization domain, OD (AA325-356), N = 4 (5.6%); C-terminal domain, CTD (AA357-393), N = 1 (1.4); Fig. 3].

### Immunohistochemical staining for p53

The most common staining pattern was the overexpression pattern (41/92, 44.6%), and 7 of 92 cases (7.6%) had a cytoplasmic pattern. In addition, five cases showed a null-pattern without nuclear overexpression. Finally, the wild-type pattern was seen in 39 of 92 cases (42.4%). Inter-rater variability for assessment of p53 expression by independent observers showed very good agreement. In addition, to avoid disagreement, no subclonal *TP53* mutations, which are thought to be one of the causes of discordances between p53 IHC expression [12] or *TP53* mutations were included in the present study, given that, as mentioned above, assessment of tumor tissue with subclonal p53 expression was not targeted to areas with mutant pattern p53 staining. For cases in which we detected discordant p53 IHC and *TP53* sequencing results, PCR-based sequencing was performed on tumor DNA extracted from the corresponding isolated tumor samples.

### Agreement of p53 IHC with the presence or absence of TP53 mutations

In samples with wild-type p53 expression, no *TP53* mutation was detected with NGS in 33 of 39 cases (84.6%), pathological missense mutations were found in six cases (15.4%). The concordance between the p53 overexpression pattern and *TP53* mutation status was high (concordant cases: 35/41 cases, 85.4%). Perfect concordance between the null-type expression pattern and *TP53* mutation status (all variants were a loss-of-function variant) was found (5 mutation variants of 5 null-type expression samples). In addition, there was one discordant case between the cytoplasmic staining pattern and *TP53* mutation status in 7 cases of cytoplasmic pattern (this expression pattern was a loss-of-function variant in 6 cases). Finally, although we validated the discordant case in the IHC staining pattern by assessing NGS sequence and Sanger sequence, no difference was detected between NGS and Sanger sequences.

### Sensitivity and specificity for the predictive value of p53 IHC expression and TP53 mutations

The concordance rate between p53 IHC patterns and *TP53* mutation status is depicted in supplementary Table 1. The sensitivity and specificity were 89.5% and 94.3%, respectively. In addition, the positive and negative predictive values were 96.2% and 84.6%, respectively. Finally, the positive likelihood ratio and negative likelihood ratio were 15.7 and 0.11, respectively. To examine the correlation between p53 IHC patterns and the corresponding *TP53* mutation type, we hypothesized that 1) the cytoplasmic pattern occurs with mutations that affect the *TP53* nuclear localization signal (amino acids 291 to 324) and oligomerization domain (amino acids 325 to 356), 2) the overexpression pattern, defined as uniformly strong staining of > 80% tumor cell nuclei, occurs with missense mutations of the DNA binding domain, 3) the null-pattern, defined as the complete absence of any staining of tumor cell nuclei, occurs with loss-of-function mutations (non-sense, frameshift, and splicing mutations) [14]. Using p53 IHC patterns to predict these mutations was relatively accurate, as shown in Table 2.

**Table 2:** Assessment of IHC pattern predicted from *TP53* mutation status

IHC expression pattern		IHC staining pattern inferred from *TP53* mutation status								kappa coefficient [95%CI]	Sensitivity	Specificity	Positive predictive value	Negative predictive value	Positive likelihood ratio	Negative likelihood ratio
		Wild (%)		Overexpression (%)		Null (%)		Cytoplasmic (%)								
Wild	39	33	(84.6)	5	(12.8)	0	(0)	1	(2.6)	0.820 [0.701, 0.938]	0.943	0.895	0.846	0.962	8.957	0.064
Overexpression	41	1	(2.4)	35	(85.4)	4	(9.8)	1	(2.4)	0.757 [0.623, 0.892]	0.875	0.885	0.854	0.902	7.583	0.141
Null	5	0	(0)	0	(0)	5	(100)	0	(0)	0.693 [0.413, 0.973]	0.556	1.000	1.000	0.954	INF	0.444
Cytoplasmic	7	1	(14.3)	0	(0)	0	(0)	6	(85.7)	0.782 [0.544, 1.000]	0.75	0.988	0.857	0.976	63.000	0.253

INF, infinity

## Discussion

In the present study, we compared p53 IHC expression with the mutation status of *TP53* as determined by NGS in patients with CRC. In the 92 samples analyzed, *TP53* mutations were detected in 72 cases (62.0%), with 53 (73.6%) variants harboring a missense variation and 19 (26.4%) variants having non-sense or frameshift mutations, including 10 stop codon variants. There were five frameshift variants, two in-frame deletions, one in-frame insertion, and one splice donor variant. The frequency of *TP53* mutations examined in the present study was consistent with previous studies [19–23]. Therefore, we believe that the present study provides reliable data on *TP53* mutations. Based on a previous study, the cut-off value for p53 IHC expression reflecting missense mutations was 80%, and the cut-off value for nonsense/frameshift mutations was 0% in the present study [15]. In addition, expression intensity and distribution were important for p53 IHC expression pattern classification. However, there may be serious limitations to this cut-off value, given that it is unclear how < 80% p53 IHC expression should be classified. For example, a case with 70% p53 IHC expression, with a diffuse and strong staining pattern, is difficult to assign into any of the four-tier classifications. However, there is no such case in the present study. Further consideration may be required for this cut-off value.

Previous studies have reported on using IHC staining for p53 as a tool to assess *TP53* mutation status [12–14, 23]. However, after the introduction of NGS, sequencing of the *TP53* gene in cancer cells has increasingly been used [9, 14]. Previous studies have shown the correlation between p53 IHC expression patterns and *TP53* variants status detected by NGS [9, 14]. Köbel et al. adopted a three-tier classification, consisting of wild-type, overexpression, and complete absence [14]. According to this report, the p53 IHC expression patterns showed excellent concordance with the *TP53* variant status [14]. The sensitivity of IHC for detecting gain-of-function variations, loss-of-function variations, and the wild-type expression of p53 was 100, 76, and 100%, respectively [13]. In addition, the specificity of IHC for detecting gain-of-function variations, loss-of-function variations, and wild-type expression of p53 was 95, 100, and 96%, respectively [13]. Recent studies have shown that p53 IHC expression patterns can be categorized as wild-type patterns or aberrant-type patterns, and this classification was found to be significantly correlated with *TP53* NGS classification in CRC [9, 13]. In gastric cancer, IHC p53 expression patterns were significantly correlated with *TP53* variations detected by NGS [24]. In the present study, we adopted a four-tier classification that categorizes samples into wild-type, overexpression, null-type, and cytoplasmic p53 patterns. We found that this classification could predict *TP53* mutation patterns with high sensitivity and specificity. Our data suggest that p53 IHC patterns have a good predictive value for *TP53* mutations.

Weakly-stained wild-type cases have frequently been misclassified, resulting in false positive mutation predictions [14]. Weakly-stained assays performed without intrinsic controls cannot reliably distinguish null-type from wild-type phenotype [14]. Therefore, we propose the use of intrinsic controls as an internal reference for IHC scoring. Despite a common consensus that p53 IHC cannot detect p53 wild-type protein due to its rapid degradation, the DO7 antibody used in this study detects p53 expression in normal cells, including stromal fibroblasts and lymphocytes, when used with recently improved polymer-based IHC detection systems [25]. It is possible that p53-positive intraepithelial lymphocytes in a complete absence case could be falsely assessed as p53 wild-type tumor cells [24]. Use of improved polymer-based IHC detection systems is needed to differentiate wild-type from null-type p53 expression [25]. Our data strongly support the contention that further assay comparison and training in interpretation are required for p53 IHC to be used as a diagnostic and predictive test.

It should be noted that the p53 cytoplasmic pattern was not a single pattern but presented as combinations of cytoplasmic and nuclear staining in a previous study [26]. The cytoplasmic staining pattern shows a spectrum of cytoplasmic stain intensity from weak to strong and from heterogeneous to uniform [26]. Importantly, if a range of nuclear expression patterns of varying intensity involving a few cells to < 80% of tumor cells was observed, such cases were classified into the p53 cytoplasmic pattern. Moreover, a whole slide section from which TMA cores were taken showed a variable amount of nuclear staining with the cytoplasmic staining in this study, supported by previous studies [14, 15]. Focal nuclear staining in tumors with p53 cytoplasmic patterns may be challenging to detect with TMAs rather than by whole slide sections [26]. This may explain some of the differences between previous studies and our data [26]. Finally, determining the prognostic value of the cytoplasmic pattern is very important, and further studies will be needed to evaluate its clinical significance.

There are some limitations to our study. First, the sample size may be too small to identify a correlation between p53 IHC patterns and *TP53* mutation status. However, given that the present sample size was higher than the minimum sample size we determined in our sample size calculations, we believe that this sample size is sufficient to investigate the association between p53 IHC patterns and *TP53* mutation status. Second, we did not examine the association of p53 IHC patterns with patient prognosis, but aim to do so in a subsequent study. In addition, we excluded CRC samples with microsatellite instability (MSI). Therefore, the present cohort is not representative for evaluating molecular alterations of CRC with such a phenotype. Finally, the p53 IHC pattern and the *TP53* mutation status were not obtained from the same site in the present study. Therefore, the p53 IHC pattern may not reflect the *TP53* mutation status. However, both sampling sites were obtained from adjacent areas. Moreover, we used isolated tumor gland samples to increase the data accuracy [18]. We suggest that the p53 IHC pattern reflects the *TP53* mutation status in the present study.

In conclusion, to our knowledge, this study is the first to examine the correlation of the proposed p53 IHC patterns with *TP53* mutation status in CRC. We revealed that the interpretation of p53 IHC patterns is highly reliable and reproducible, and can serve as an excellent surrogate approach for assigning p53 IHC classes. In addition, we showed a high agreement supported by optimal laboratory protocols with adequate controls. Experience, training, and proper p53 IHC staining protocols will be required for routine diagnostic pathology. Nevertheless, the combination of p53 IHC and sequencing should be helpful in considering the p53 functional status for clinical applications.

## Supplementary Information

Below is the link to the electronic supplementary material.

Supplementary file1 (DOCX 13 KB)

## Data Availability

The data that support the findings of our study are available from the corresponding author upon reasonable request.
