# Supplementary material for: The pattern-based interpretation of p53 immunohistochemical expression as a surrogate marker for TP53 mutations in colorectal cancer
Source: Virchows Arch. 2024 Mar 21;486(2):333–41. doi: 10.1007/s00428-024-03790-z (PMC11876225; doi:10.1007/s00428-024-03790-z)
Supplement: Supplementary file 1 — Supplementary file1 (DOCX 13 KB) [file 428_2024_3790_MOESM1_ESM.docx]

Supplementary Table 1. Measurement of the sensitivity and specificity for concordance of immunohistochemical expression and TP53 mutation status

| Sensitivity | Specificity | Positive  predictive  value | Negative  predictive  value | Positive  likelihood  ratio | Negative  likelihood  ratio |
| --- | --- | --- | --- | --- | --- |
| 0.895 | 0.943 | 0.962 | 0.846 | 15.658 | 0.112 |
